# Prevention of bicalutamide-induced breast events in patients with prostate cancer: a meta-analysis of randomized controlled trials

**DOI:** 10.1007/s40618-025-02583-8

**Published:** 2025-04-17

**Authors:** Luca Spagnolo, Daniele Tienforti, Carolina Moretto, Camilla Tonni, Vittoria Donatelli, Alessandro Ferranti, Gennaro Puocci, Claudio Capuano, Arcangelo Barbonetti

**Affiliations:** 1 Andrology Unit, Department of Clinical Medicine, Life, Health and Environmental Sciences, University of L’Aquila, L’Aquila, Italy; 2 Spinal Unit, San Raffaele Sulmona Institute, Sulmona, Italy; 3 Department of Veterinary Medicine, University of Teramo, Teramo, Italy; 4 Department of Clinical Medicine, Life, Health and Environmental Sciences, University of L’Aquila, L’Aquila, 67100 Italy

**Keywords:** Aromatase, Bicalutamide, Gynecomastia, Radiotherapy, Tamoxifen, Testosterone

## Abstract

**Purpose:**

This study aimed to quantitatively assess the effectiveness of tamoxifen, anastrozole, and radiotherapy in preventing bicalutamide-induced breast events—specifically gynecomastia and breast pain—in patients with prostate cancer.

**Methods:**

A systematic review and meta-analysis of randomized controlled trials (RCTs) was conducted according to PRISMA-P guidelines. A comprehensive search was performed in PubMed, Scopus, and Web of Science for English-language studies without temporal restrictions. Studies were included if they involved prostate cancer patients treated with bicalutamide receiving preventive interventions (tamoxifen, anastrozole, or radiotherapy) compared to bicalutamide alone (or bicalutamide plus placebo/sham). Data extraction focused on the incidence of gynecomastia and breast pain, and study quality was assessed using the Jadad scale. Risk ratios (RR) with 95% confidence intervals (CI) were calculated using fixed- or random-effects models, and heterogeneity was evaluated with the I² statistic. Publication bias was explored via funnel plots and the trim-and-fill method.

**Results:**

Nine RCTs met the inclusion criteria. Tamoxifen significantly reduced the risk of breast events by 82% (RR: 0.18, 95% CI: 0.08–0.38 for gynecomastia and RR: 0.18, 95% CI: 0.07–0.43 for breast pain). Radiotherapy reduced gynecomastia risk by 52% (RR: 0.48, 95% CI: 0.38–0.59) and breast pain by 34% (RR: 0.66, 95% CI: 0.48–0.90). Anastrozole did not show significant benefit.

**Conclusion:**

Tamoxifen appears to be the most effective strategy for preventing bicalutamide-induced breast events, with radiotherapy serving as a viable alternative, and anastrozole offering no benefit. Further large-scale, high-quality studies are needed to confirm these findings and refine preventive treatment recommendations.

## Introduction

Prostate cancer is a multi-stage disease that exists as a continuum of several, partially overlapping stages, including localized, locally advanced, metastatic, and hormone-refractory forms [1, 2]. The common therapeutic approaches in early stages include radical prostatectomy, radiotherapy, and active monitoring [3, 4]. Bicalutamide is a potent non-steroidal antiandrogen administered at 150 mg per day. It was historically considered a treatment option for patients with locally advanced, non-metastatic (M0) prostate cancer [5–11]. However, current National Comprehensive Cancer Network (NCCN) [12] and European Society for Medical Oncology (ESMO) [13] guidelines no longer recommend it as a first-line therapy, favoring next-generation androgen receptor pathway inhibitors (ARPIs) (e.g., enzalutamide, apalutamide, darolutamide) due to their superior efficacy and safety profile. Nevertheless, the guidelines form the European Association of Urology (EAU) [14] still recognize a role for bicalutamide in certain clinical scenarios, particularly for patients who cannot tolerate next-generation ARPIs. Importantly, while enzalutamide monotherapy has been associated with a 44.9% incidence of gynaecomastia [15], bicalutamide remains a well-documented cause of breast-related side effects, including gynecomastia and breast pain [7–11]. Gynecomastia is a benign overgrowth of glandular tissue in the male breast induced by an imbalance in sex hormones: while decreasing androgen levels should inhibit breast enlargement, increased estrogen levels promote mammary glandular development [16, 17]. Gynecomastia progresses through distinct stages. In its initial phase, which may last up to two years, the condition is characterized by a “proliferative” process involving both ductal cells and fibroblastic-stromal tissue [18]. This phase is frequently accompanied by pain and breast tenderness, although gynecomastia may eventually present as an isolated symptom without pain [19]. Over time, the condition may become chronic and irreversible due to stromal hyalinization and fibrosis [20].

Bicalutamide interferes at multiple levels with the hormonal balance that regulates the proliferative state of mammary glandular tissue in males [21]. As an androgen receptor antagonist, it diminishes the antiproliferative effects of testosterone while simultaneously inducing a compensatory increase in testosterone levels that are aromatized into 17-beta-estradiol [22]. An increased estrogen/androgen ratio underlies the pathophysiology of breast complications associated with bicalutamide therapy [17, 23]. On this basis, pharmacological strategies to prevent breast enlargement aim to block the estrogen-specific stimulation of mammary tissue and/or to limit the peripheral aromatization of testosterone. Anti-estrogens, such as tamoxifen, and aromatase inhibitors, such as anastrozole, have been employed for these purposes [1, 6, 24–27]. Low-dose irradiation therapy, by reducing the responsiveness of mammary tissue to hormonal stimulation, represents another non-surgical strategy to mitigate the effects of bicalutamide on the breast [24, 28–31]. Although the literature documents the efficacy of some approaches– including tamoxifen treatment– the small sample sizes do not allow for definitive conclusions. For other strategies, such as treatment with aromatase inhibitors and radiotherapy, the evidence remains inconclusive [32].

The aim of our study is to quantitatively assess the effectiveness of tamoxifen, anastrozole, and radiotherapy in preventing bicalutamide-induced gynecomastia and breast pain in patients with prostate cancer through a systematic review and meta-analysis of randomized controlled trials (RCTs).

## Materials and methods

The study was conducted in accordance with the Preferred Reporting Items for Systematic Review and Meta-Analysis Protocols (PRISMA-P) [33]. The study is registered in the PROSPERO (International Prospective Register of Systematic Reviews) database under the identification number CRD42023484338.

## Systematic search strategy

A systematic search was performed in PubMed, Scopus, and Web of Science. The selection was restricted to studies published in English, with no temporal limits. The following search terms were used: “gynecomastia,” “breast enlargement,” “mastodynia,” “breast pain,” “breast event*,” “bicalutamide,” “antiandrogen*,” “SERM*,” “selective estrogen receptor modulator*,” “tamoxifen,” “clomiphene,” “aromatase inhibitor*,” “aromatase inhibition,” “anastrozole,” “radiotherapy,” and “breast irradiation.” Boolean AND/OR operators were applied to combine these terms appropriately.

## Inclusion/exclusion criteria

The study selection proceeded in several steps. First, a comprehensive identification of articles on the topic was achieved through database searches. After duplicate removal, titles and abstracts were screened. Finally, two reviewers (D.T. and C.M.) independently assessed the full texts of the remaining articles to verify eligibility. Only studies meeting the following criteria were included: (1) RCTs; (2) studies conducted in patients with prostate cancer treated with bicalutamide; (3) studies in which patients received a preventive treatment regimen with tamoxifen, anastrozole, or radiation therapy; (4) studies that compared the preventive treatment group with a control group receiving bicalutamide alone (or bicalutamide plus placebo/sham) in terms of bicalutamide-induced breast events (gynecomastia and/or breast pain).

## Data extraction

For each selected study, the following information was extracted by two authors (L.S. and D.T.): the first author’s name, publication year, the number of patients experiencing the event(s) (gynecomastia and/or breast pain) in the “bicalutamide with preventive treatment” group versus the group bicalutamide alone (or bicalutamide plus placebo/sham)”, and the total number of patients in both groups.

## Quality assessment

The methodological quality of each study was evaluated using the Jadad scale [34], a validated instrument that assesses randomization, double blinding, and losses to follow-up on a scale from 0 to 5. A score of ≥ 3 was considered indicative of good methodological quality. Two authors (A.F. and G.P.) evaluated the quality of each selected study, involving a third author (A.B.) to solve any disagreement.

### Statistical analysis

The efficacy of each intervention (tamoxifen, anastrozole, and radiotherapy) in preventing bicalutamide-induced adverse breast events was assessed by calculating risk ratios (RR) with 95% confidence intervals (95% CIs). Data were pooled using random-effects models even in the presence of minimal heterogeneity; a fixed-effects model was applied only when no heterogeneity was detected. Between-study heterogeneity was evaluated using Cochran’s Chi-square test (Q test) and the I² statistic, with I² ≥ 50% and/or a P value < 0.05 considered indicative of significant heterogeneity [35]. Sub-analyses were performed to explore potential sources of heterogeneity. Publication bias was examined by inspecting the symmetry of funnel plots [36]. To adjust for potential publication bias, Duval and Tweedie’s “trim-and-fill” method was applied [37], which identifies putative missing studies to rebalance the distribution and yields an adjusted pooled estimate. Analyses were conducted using RevMan (Review Manager, version 5.3) and R statistical software with the metafor package (version 3.6.3, 2020; The R Foundation for Statistical Computing, Vienna, Austria).

## Results

### Study selection

**Fig. 1:**
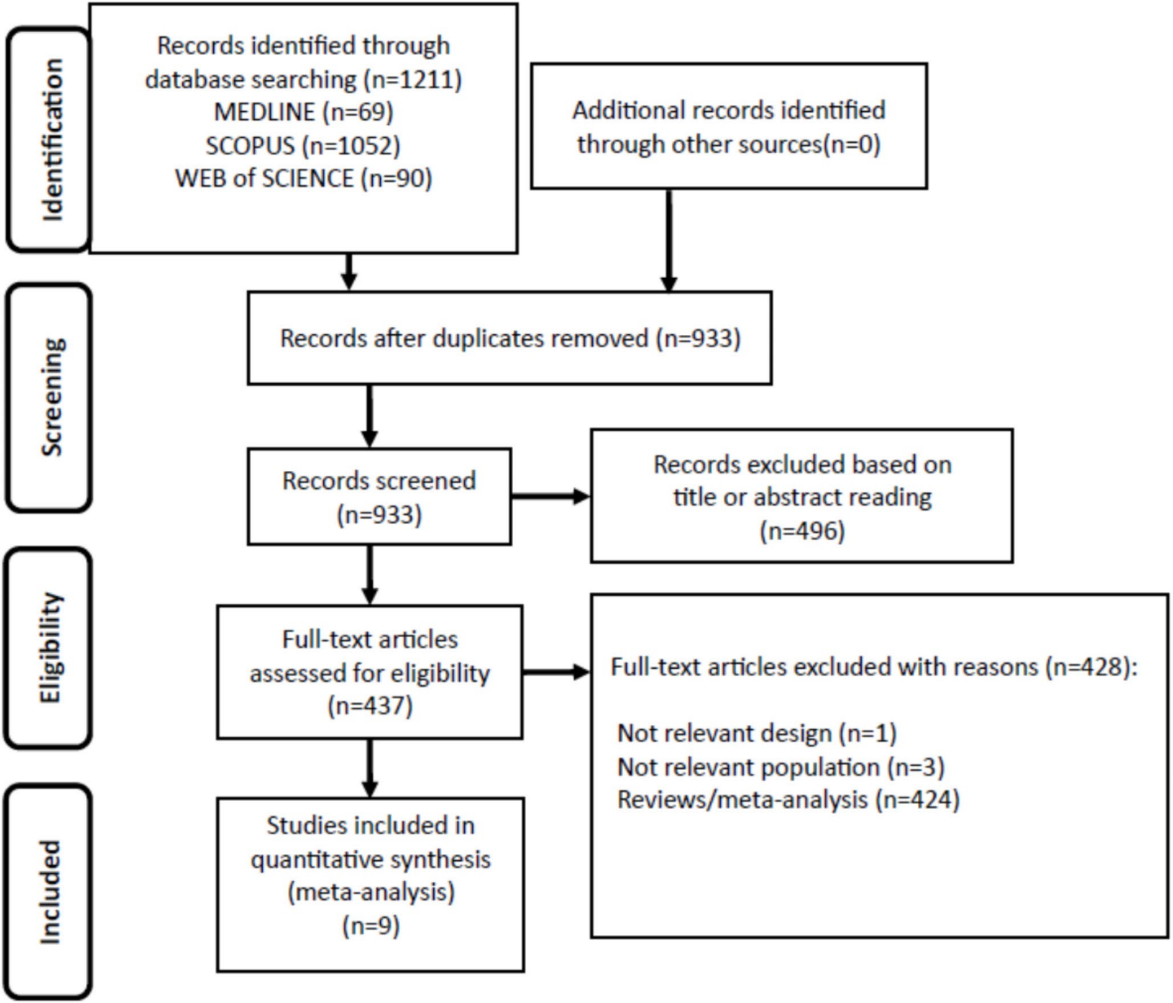
Flow diagram showing an overview of the study selection process

The online database search initially retrieved 1211 studies. After removing duplicates, 933 studies remained. Following the screening of titles and abstracts, 496 studies were excluded as not relevant. Thus, as shown in Fig. 1, after further full text evaluation, 9 studies met the inclusion criteria [1, 6, 24–30]. The main details of the eligible studies, including their methodological quality scores according to the Jadad scale, are presented in Table 1. With the exception of one RCT [30], all studies were assigned a score of ≥ 3.

**Table 1:** Main characteristics of the RCTs included in the meta-analysis and their methodological quality scores according to the Jadad scale

Study	Country	Interventions	Gynecomastia assessment	Breast pain assessment	Follow-up (months)	Jadad score*
Widmark et al., 2003	Norway	Bica alone (*n* = 79) or with RT 12 to 15 Gy in a single fraction or 15 Gy in 3 fractions (*n* = 174) on a target between 4–6 cm diameter	Physical examination, self-reported, PCSS questionnaire	Physical examination, self-reported, PCSS questionnaire	12	2
Tyrrell et al., 2004	Sweden and UK	Bica with sham (*n* = 54) or with a single 10 Gy RT dose (*n* = 52) on a 5 cm diameter circle of tissue around the nipple	Callipers	Self-reported	12	4
Boccardo et al., 2005	Italy	Bica with Placebo (*n* = 40) or Tam 20 mg daily (*n* = 37) or Anas 1 mg daily (*n* = 36)	Callipers or ultrasound	Self-reported	Median: 12 (range: 1 to 25)	4
Di Lorenzo et al., 2005	Italy	Bica alone (*n* = 33) or with Tam 10 mg daily (*n* = 34) or a single 12 Gy RT dose (*n* = 35) on a 5 cm diameter circle of tissue around the nipple	Callipers	Self-reported	Median 26 (range: 13 to 35)	3
Perdonà et al., 2005	Italy	Bica alone (*n* = 51) or with Tam 10 mg daily (*n* = 50) or a single 12 Gy RT dose (*n* = 50) on a 5 cm diameter circle of tissue around the nipple	Callipers	Self-reported	12	3
Saltzstein et al., 2005	USA	Bica with Placebo (*n* = 36) or Tam 20 mg daily (*n* = 34) or Anas 1 mg daily (*n* = 36)	Physical examination and a questionnaire	Questionnaire	9	5
Fradet et al., 2007	Canada, Finland, Norway and UK	Bica with Placebo (*n* = 60) or Tam 1 (*n* = 58), 2.5 (*n* = 45), 5 (*n* = 47), 10 (*n* = 34) or 20 mg (*n* = 34)	Callipers	Self-reported	12	5
Ozen et al., 2010	Turkey	Bica alone (*n* = 61) or with RT 12 Gy dose in 2 fractions of 6 Gy each on consecutive days (*n* = 44) on a 5 cm diameter circle of tissue around the nipple	Callipers	Self-reported	12	3
Serretta et al., 2012	Italy	Bica alone (*n* = 83) or with Tam 10 mg daily (*n* = 80)	Self-administered visual analogue scale	Self-administered visual analogue scale	Median 15 (range: 12 to 36)	3

*A Jadad score ≥ 3 documented a good methodological quality. Anas, anastrozole; Bica, bicalutamide; Gy, Gray; PCSS, Prostate Cancer Symptom Scale; Tam, tamoxifen; RT, radiotherapy

### Tamoxifen in prevention of bicalutamide-induced gynecomastia

**Fig. 2:**
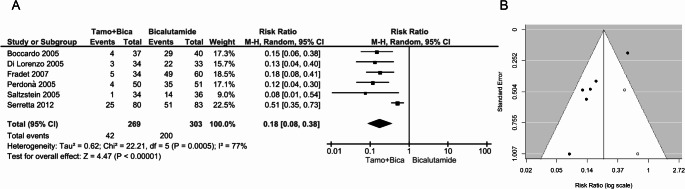
(**A**) Forest plot and (**B**) funnel plot depicting the effect of tamoxifen in preventing bicalutamide-induced gynecomastia. In panel A, the diamond represents the overall estimate (with the width indicating the 95% CI), and the boxes represent the weight of individual studies in the pooled result. In panel B, the trim-and-fill test identifies two putative missing studies (white circles) on the right side of the distribution

Six RCTs provided data on 572 patients treated with bicalutamide, of whom 269 also received tamoxifen. As shown in Fig. 2A, the pooled estimate revealed an 82% risk reduction of gynecomastia in the tamoxifen-treated group (RR: 0.18, 95% CI: 0.08–0.38, *p* < 0.00001). However, significant between-study heterogeneity was observed (I² = 77%, *p* = 0.0005). The skewed funnel plot suggested publication bias, which was confirmed by the trim-and-fill test that identified two putative missing studies (Fig. 2B). Nevertheless, publication bias did not fully explain the heterogeneity, as the overall estimate recalculated after the inclusion of the two putative missing studies still revealed significant heterogeneity (adjusted pooled RR: 0.23, 95% CI: 0.13–0.39; I² = 60%, *p* = 0.004). In contrast, when the analysis was restricted to the three double-blind, randomized, placebo-controlled trials [6, 25, 26], the overall estimate remained substantially unchanged (RR: 0.15, 95% CI: 0.08–0.27, *p* < 0.00001 with fixed-effects model) and heterogeneity disappeared (I² = 0%, *p* = 0.72).

### Tamoxifen in prevention of bicalutamide-induced breast pain

**Fig. 3:**
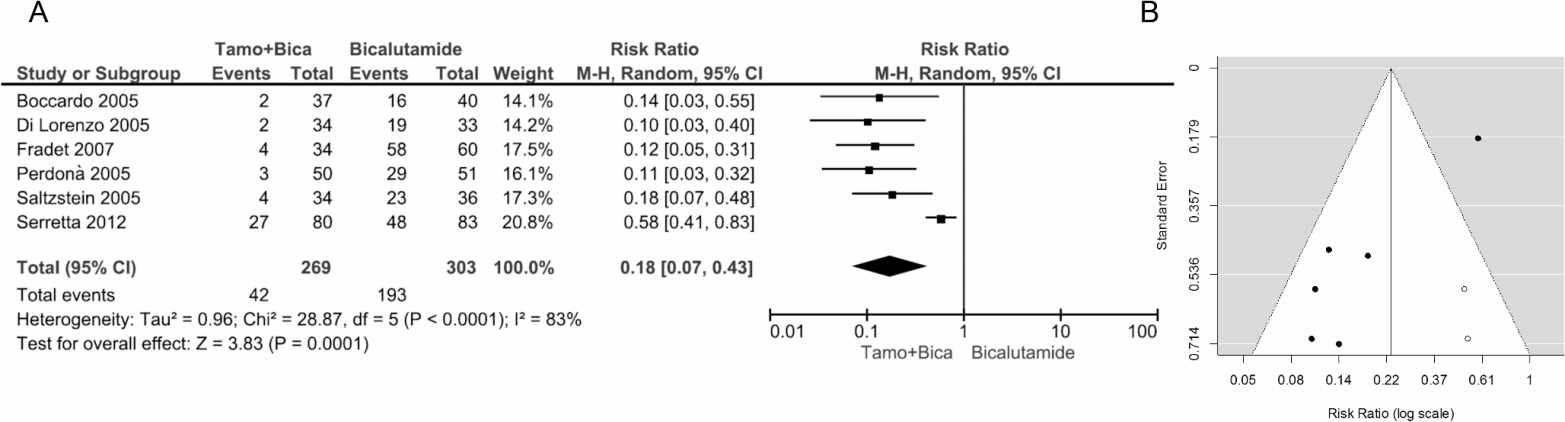
(**A**) Forest plot and (**B**) funnel plot depicting the effect of tamoxifen in preventing bicalutamide-induced breast pain. In panel A, the diamond represents the overall estimate (with the width indicating the 95% CI), and the boxes represent the weight of individual studies in the pooled result. In panel B, the trim-and-fill test identifies two putative missing studies (white circles) on the right side of the distribution

As shown in Fig. 3A, tamoxifen treatment demonstrated significant efficacy in preventing bicalutamide-induced breast pain (RR: 0.18, 95% CI: 0.07–0.43, *p* = 0.00001). However, significant between-study heterogeneity was registered (I² = 83%, *p* < 0.0001). The skewed funnel plot indicated publication bias, which was confirmed by the trim-and-fill test that identified two putative missing studies (Fig. 3B). Nonetheless, publication bias did not fully account for the heterogeneity, as the overall estimate recalculated after the inclusion of the putative missing studies still showed significant heterogeneity (adjusted pooled RR: 0.23, 95% CI: 0.13-0.42; I² =65%, *p=*0.0009). On the contrary, when the analysis was restricted to the three double-blind, randomized, placebo-controlled trials [6, 25, 26], the overall estimate remained largely unchanged (RR: 0.15, 95% CI: 0.08-0.27, *p* < 0.00001 with fixed-effects model) with no between-study heterogeneity (I² = 0%, *p* = 0.82).

### Radiotherapy in prevention of bicalutamide-induced gynecomastia

**Fig. 4:**
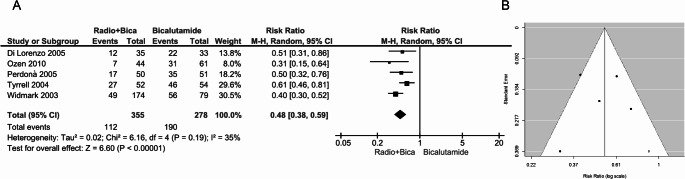
(**A**) Forest plot and (**B**) funnel plot depicting the effect of radiotherapy in preventing bicalutamide-induced gynecomastia. The diamond represents the overall estimate (with the width indicating the 95% CI), and the boxes represent the weight of individual studies in the pooled result. In panel B, the trim-and-fill test identifies one putative missing study (white circle) on the right side of the distribution

Five RCTs provided data on 633 patients treated with bicalutamide, of whom 355 also underwent radiotherapy. Notably, only the RCT by Tyrrell [29] was a double-blind, sham-controlled trial. As shown in Fig. 4A, the pooled estimate indicated a 52% risk reduction of gynecomastia in the radiotherapy-treated group (RR: 0.48, 95% CI: 0.38–0.59, *p* < 0.00001) with non-significant heterogeneity (I² = 35%, *p* = 0.19). The trim-and-fill test identified one putative missing study (Fig. 4B), the inclusion of which, did not affect the overall estimate (adjusted pooled RR: 0.53, 95%CI: 0.41-0.86, p<0.0001).

### Radiotherapy in prevention of bicalutamide-induced breast pain

**Fig. 5:**
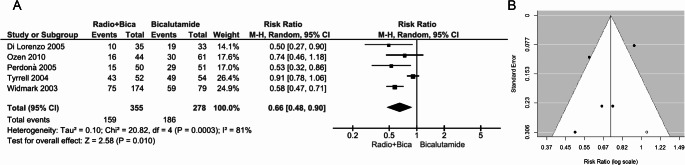
(**A**) Forest plot and (**B**) funnel plot depicting the effect of radiotherapy in preventing bicalutamide-induced breast pain. In panel A, the diamond represents the overall estimate (with the width indicating the 95% CI), and the boxes represent the weight of individual studies in the pooled result. In panel B, the trim-and-fill test identifies one putative missing study (white circle) on the right side of the distribution

As shown in Fig. 5A, the overall efficacy of radiotherapy in preventing bicalutamide-induced breast pain reached statistical significance (RR: 0.66, 95% CI: 0.48–0.90, *p* = 0.01). However, significant between-study heterogeneity was observed (I² = 81%, *p* = 0.0003). The skewed funnel plot indicated publication bias, which was confirmed by the trim-and-fill test that identified one putative missing study (Fig. 5B). Nevertheless, publication bias did not completely account for the heterogeneity, as the overall estimate recalculated after adjustment for the putative missing study still revealed significant heterogeneity (adjusted pooled RR: 0.73, 95% CI: 0.58–0.92; I² = 63.6%, *p* = 0.004).

### Anastrozole in prevention of bicalutamide-induced breast events

The potential usefulness of anastrozole in preventing bicalutamide-induced breast events was evaluated in only two RCTs, both of which were double-blind, placebo-controlled. These studies provided data on a total of 148 patients treated with bicalutamide, of whom 72 also received anastrozole. The pooled estimates demonstrated that anastrozole was ineffective in preventing both bicalutamide-induced gynecomastia (RR: 0.75, 95% CI: 0.54–1.04, *p* = 0.08; I² = 0%, *p* = 0.55) and breast pain (RR: 0.88, 95% CI: 0.64–1.21, *p* = 0.08; I² = 4%, *p* = 0.31).

## Discussion

Although bicalutamide has historically played a role in the management of prostate cancer, its clinical relevance has declined with the advent of more effective androgen receptor inhibitors. While NCCN [12] and ESMO guidelines [38] no longer recommend it as a preferred option, EAU guidelines [39] still consider it a viable alternative in specific cases. However, a key concern associated with bicalutamide use is its high incidence of gynaecomastia [7–11], which can significantly impact patient adherence and quality of life. This adverse effect primarily results from its antagonistic action on androgen receptors in breast tissue, leading to an imbalance in the estrogen–androgen ratio and, consequently, to the stimulation of breast tissue growth [10]. Unlike recent studies that primarily focus on enzalutamide-induced breast-related side effects, our study directly addresses the management of bicalutamide-induced gynaecomastia, which remains an underexplored yet clinically relevant issue. Recently, Tsuboi et al. [40] provided a broad analysis of breast-related side effects in antiandrogen therapy and assessed the prevention of gynecomastia and breast pain in patients undergoing antiandrogen therapy for prostate cancer. Nevertheless, their meta-analysis did evaluate aromatase inhibitors as a preventive or therapeutic strategy. By addressing this gap, our study offers a more comprehensive and targeted approach to managing this well-recognized adverse effect.

The results of this meta-analysis highlight the efficacy of specific strategies—such as tamoxifen and radiotherapy—while demonstrating the limited utility of anastrozole in mitigating these adverse events. Nevertheless, the small number of available trials on the efficacy of anastrozole prevents definitive conclusions.

Although tamoxifen has not yet been approved by the Food and Drug Administration (FDA) for this indication, our meta-analysis demonstrated its robust efficacy in preventing both gynecomastia and breast pain associated with bicalutamide therapy. Pooled estimates from six RCTs indicated that tamoxifen reduced the risk of breast events by 82%. Despite significant heterogeneity across studies—particularly in the analysis of gynecomastia prevention—sensitivity analyses restricted to double-blind, placebo-controlled RCTs yielded consistent results and eliminated heterogeneity. These findings are consistent with tamoxifen’s established role as a selective estrogen receptor modulator (SERM), which competes with estrogen for receptor binding in mammary tissue and effectively mitigates estrogenic stimulation [6]. Notably, its ability to block estrogen receptors in the breast while acting as an estrogen agonist in other tissues (such as bone) provides a therapeutic advantage in managing gynecomastia without adversely affecting other estrogen-dependent tissues [41]. Although the asymmetry observed in the funnel plots of tamoxifen analyses suggested the presence of publication bias, adjusted RRs using the trim-and-fill method confirmed the robustness of the results.

Radiotherapy, particularly low-dose breast irradiation, is another modality employed to prevent or manage gynecomastia. This approach induces fibrosis and tissue atrophy, thereby limiting the proliferative effects of estrogen on breast tissue [29, 42]. In our analysis, radiotherapy emerged as an effective strategy, reducing the risk of gynecomastia by 52% and breast pain by 34%. Unlike tamoxifen, the effectiveness of radiotherapy in preventing gynecomastia was associated with minimal heterogeneity—likely due to uniform treatment protocols across studies. However, its efficacy in preventing breast pain was less pronounced and accompanied by significant heterogeneity, which persisted even after adjusting for publication bias. These findings suggest that radiotherapy may not uniformly address all aspects of bicalutamide-induced breast events, particularly subjective symptoms such as pain [30].

Anastrozole, an aromatase inhibitor, acts by inhibiting the conversion of androgens to estrogens. Given that bicalutamide indirectly increases estrogen levels by reducing androgenic feedback on the hypothalamic–pituitary–gonadal axis, anastrozole could theoretically reduce estrogen production and attenuate its stimulatory effects on breast tissue [43]. In contrast to tamoxifen and radiotherapy, however, our meta-analysis did not demonstrate significant efficacy for anastrozole in preventing either gynecomastia or breast pain. This lack of efficacy, arising from the results of only two RCTs, may reflect insufficient suppression of estrogen levels needed to counteract the hormonal imbalances induced by bicalutamide, which aligns with the limited and inconsistent evidence available for aromatase inhibitors in this setting [6, 25].

Our findings have several clinical implications. First, tamoxifen should be considered the first-line preventive treatment for bicalutamide-induced breast events given its superior efficacy and consistent results across trials. Radiotherapy represents a viable alternative, particularly for patients who are intolerant to tamoxifen or in cases where preventing gynecomastia is prioritized over managing breast pain. Conversely, the lack of benefit observed with anastrozole suggests that its routine use in this setting is unwarranted. Furthermore, while both tamoxifen and radiotherapy demonstrated efficacy, the heterogeneity observed in some analyses highlights the importance of tailoring preventive strategies to individual patient profiles. Factors such as baseline hormonal levels, comorbidities, breast tissue responsiveness, and patient preferences should guide treatment decisions. Finally, the choice between tamoxifen and radiotherapy should also take into account potential side effects. Systemic effects of tamoxifen may include hot flashes [24, 25], an increased risk of cardiovascular events [27], which may be particularly relevant in older patients with cardiovascular comorbidities. On the other hand, prophylactic radiotherapy is a localized intervention, generally well tolerated, but associated with potential short-term skin irritation, nipple tenderness, mild discomfort, and pruritus at the treatment site [1, 24, 28, 29].

This study also has limitations. Although all RCTs on tamoxifen consistently indicate its efficacy in preventing both bicalutamide-induced gynecomastia and breast pain, the overall quality of the evidence is limited by the low number of RCTs, small sample sizes, and the relatively wide confidence intervals of documented outcomes. These factors limit the statistical power of the overall effect estimates. Additionally, potential attrition bias exists, as not all RCTs adequately reported dropout rates. Significant heterogeneity was observed among the RCTs—a heterogeneity that was not explained by publication bias but was eliminated when the three non-double-blind, placebo-controlled trials were excluded. This heterogeneity likely also reflects differences in tamoxifen dosing (10 mg versus 20 mg daily) and treatment duration (ranging from 3 to 12 months). In the absence of comparative studies, no definitive recommendation can be made regarding the optimal dosage. In an open multicenter randomized phase 3 trial [44], daily treatment with tamoxifen 20 mg was superior to a regimen involving tamoxifen 20 mg weekly (after an initial 8-week period of daily therapy) in preventing bicalutamide-induced gynecomastia and breast pain. Regarding anastrozole, the two available RCTs [6, 26] consistently reported its ineffectiveness in preventing bicalutamide-induced breast events. Overall, the small sample sizes of the included trials limit the generalizability of our findings. In contrast, radiotherapy appears to offer some prophylactic efficacy, although the availability of only one sham-controlled RCT [29], variations in doses (10 to 15 Gy), treatment modalities, and the significant heterogeneity observed in the analysis of breast pain downgrades the overall quality of the evidence.

Future studies should aim to evaluate the comparative efficacy of these preventive strategies in larger populations and explore potential combination therapies to enhance outcomes. Moreover, the long-term safety profiles of these interventions—particularly tamoxifen and radiotherapy—warrant further investigation to ensure their broader applicability in clinical practice.

In conclusion, this meta-analysis highlights tamoxifen and radiotherapy as effective strategies for preventing bicalutamide-induced breast events, with tamoxifen demonstrating superior efficacy in both gynecomastia and breast pain prevention. On the contrary, although further RCTs are warranted, anastrozole does not appear to offer significant benefits in this context. These findings underscore the need for individualized approaches to managing bicalutamide-induced adverse effects and call for further high-quality research to refine treatment recommendations.

## Data Availability

The authors confirm that the data supporting the findings of this study are available within the article.

## References

[1] Perdonà S, Autorino R, De Placido S, D’Armiento M, Gallo A, Damiano R, Pingitore D, Gallo L, De Sio M, Bianco AR, Di Lorenzo G (2005) Efficacy of tamoxifen and radiotherapy for prevention and treatment of gynaecomastia and breast pain caused by bicalutamide in prostate cancer: a randomised controlled trial. Lancet Oncol.;6(5):295–300. 10.1016/S1470-2045(05)70103-0. PMID: 15863377 10.1016/S1470-2045(05)70103-0 15863377

[2] Mallah H, Diabasana Z, Soultani S, Idoux-Gillet Y, Massfelder T (2025) Prostate cancer: A journey through its history and recent developments. Cancers (Basel) 17(2):194. 10.3390/cancers17020194PMID: 39857976; PMCID: PMC11763992 39857976 10.3390/cancers17020194 PMC11763992

[3] Hamdy FC, Donovan JL, Lane JA, Metcalfe C, Davis M, Turner EL, Martin RM, Young GJ, Walsh EI, Bryant RJ, Bollina P, Doble A, Doherty A, Gillatt D, Gnanapragasam V, Hughes O, Kockelbergh R, Kynaston H, Paul A, Paez E, Powell P, Rosario DJ, Rowe E, Mason M, Catto JWF, Peters TJ, Oxley J, Williams NJ, Staffurth J, Neal DE (2023) ProtecT Study Group. Fifteen-Year Outcomes after Monitoring, Surgery, or Radiotherapy for Prostate Cancer. N Engl J Med.;388(17):1547–1558. 10.1056/NEJMoa2214122. Epub 2023 Mar 11. PMID: 36912538 10.1056/NEJMoa2214122 36912538

[4] Wang F, Fan Y, Yin X, Qi LW, Ma G, Yuan Q (2021) Prognostic comparison between radical prostatectomy and radiotherapy in prostate cancer patients at different stages and ages. Aging 13(12):16773–16785. 10.18632/aging.203198Epub 2021 Jun 29. PMID: 34185023; PMCID: PMC8266375 34185023 10.18632/aging.203198 PMC8266375

[5] Iversen P (2002) Antiandrogen monotherapy: indications and results. Urology.;60(3 Suppl 1):64–71. 10.1016/s0090-4295(02)01576-5. PMID: 12231053 10.1016/s0090-4295(02)01576-5 12231053

[6] Boccardo F, Rubagotti A, Battaglia M, Di Tonno P, Selvaggi FP, Conti G, Comeri G, Bertaccini A, Martorana G, Galassi P, Zattoni F, Macchiarella A, Siragusa A, Muscas G, Durand F, Potenzoni D, Manganelli A, Ferraris V, Montefiore F (2005) Evaluation of tamoxifen and anastrozole in the prevention of gynecomastia and breast pain induced by bicalutamide monotherapy of prostate cancer. J Clin Oncol.;23(4):808– 15. 10.1200/JCO.2005.12.013. PMID: 15681525 10.1200/JCO.2005.12.013 15681525

[7] Cornford P, van den Bergh RCN, Briers E, Van den Broeck T, Brunckhorst O, Darraugh J, Eberli D, De Meerleer G, De Santis M, Farolfi A, Gandaglia G, Gillessen S, Grivas N, Henry AM, Lardas M, van Leenders GJLH, Liew M, Linares Espinos E, Oldenburg J, van Oort IM, Oprea-Lager DE, Ploussard G, Roberts MJ, Rouvière O, Schoots IG, Schouten N, Smith EJ, Stranne J, Wiegel T, Willemse PM, Tilki D (2024) EAU-EANM-ESTRO-ESUR-ISUP-SIOG guidelines on prostate Cancer-2024 update. Part I: screening, diagnosis, and local treatment with curative intent. Eur Urol 86(2):148–163. 10.1016/j.eururo.2024.03.027Epub 2024 Apr 13. PMID: 38614820 38614820 10.1016/j.eururo.2024.03.027

[8] See W, Iversen P, Wirth M, McLeod D, Garside L, Morris T (2003) Immediate treatment with bicalutamide 150 mg as adjuvant therapy significantly reduces the risk of PSA progression in early prostate cancer. Eur Urol.;44(5):512-7; discussion 517-8. 10.1016/s0302-2838(03)00366-x. PMID: 14572747 10.1016/s0302-2838(03)00366-x 14572747

[9] Wirth MP, See WA, McLeod DG, Iversen P, Morris T, Carroll K (2004) Casodex Early Prostate Cancer Trialists’ Group. Bicalutamide 150 mg in addition to standard care in patients with localized or locally advanced prostate cancer: results from the second analysis of the early prostate cancer program at median followup of 5.4 years. J Urol.;172(5 Pt 1):1865-70. 10.1097/01.ju.0000140159.94703.80. PMID: 15540740 10.1097/01.ju.0000140159.94703.80 15540740

[10] See WA, Wirth MP, McLeod DG, Iversen P, Klimberg I, Gleason D, Chodak G, Montie J, Tyrrell C, Wallace DM, Delaere KP, Vaage S, Tammela TL, Lukkarinen O, Persson BE, Carroll K, Kolvenbag GJ, Casodex Early Prostate Cancer Trialist Group (2002). Bicalutamide as immediate therapy either alone or as adjuvant to standard care of patients with localized or locally advanced prostate cancer: first analysis of the early prostate cancer program. J Urol.;168(2):429– 35. Erratum in: J Urol 2002;168(6):2558. Erratum in: J Urol 2002;168;4(Pt 1):1510. PMID: 12131282 12131282

[11] Iversen P, McLeod DG, See WA, Morris T, Armstrong J, Wirth MP (2010) Casodex Early Prostate Cancer Trialists’ Group. Antiandrogen monotherapy in patients with localized or locally advanced prostate cancer: final results from the bicalutamide Early Prostate Cancer programme at a median follow-up of 9.7 years. BJU Int.;105(8):1074-81. 10.1111/j.1464-410X.2010.09319.x. PMID: 22129214 10.1111/j.1464-410X.2010.09319.x 22129214

[12] National Comprehensive Cancer Network NCCN Clinical Practice Guidelines in Oncology: [Prostate Cancer] Version [3.2024]. 2024. Available online: https://www.nccn.org/professionals/physician_gls/pdf/prostate.pdf

[13] Fizazi K, Gillessen S, ESMO Guidelines Committee (2023) Electronic address: clinicalguidelines@esmo.org. Updated treatment recommendations for prostate cancer from the ESMO clinical practice guideline considering treatment intensification and use of novel systemic agents. Ann Oncol 34(6):557–563 Epub 2023 Mar 21. PMID: 36958590 36958590 10.1016/j.annonc.2023.02.015

[14] Tilki D, van den Bergh RCN, Briers E, Van den Broeck T, Brunckhorst O, Darraugh J, Eberli D, De Meerleer G, De Santis M, Farolfi A, Gandaglia G, Gillessen S, Grivas N, Henry AM, Lardas M, van Leenders JLH, Liew G, Linares Espinos M, Oldenburg E, van Oort J, Oprea-Lager IM, Ploussard DE, Roberts G, Rouvière MJ, Schoots O, Schouten IG, Smith N, Stranne EJ, Wiegel J, Willemse T, Cornford PM (2024) EAU-EANM-ESTRO-ESUR-ISUP-SIOG guidelines on prostate cancer. Part II-2024 update: treatment of relapsing and metastatic prostate cancer. Eur Urol 86(2):164–182. 10.1016/j.eururo.2024.04.010Epub 2024 Apr 29. PMID: 38688773 38688773 10.1016/j.eururo.2024.04.010

[15] Shelan M, Achard V, Appiagyei F, Mose L, Zilli T, Fankhauser CD, Zamboglou C, Mohamad O, Aebersold DM, Cathomas R (2024) Role of enzalutamide in primary and recurrent non-metastatic hormone sensitive prostate cancer: a systematic review of prospective clinical trials. Prostate Cancer Prostatic Dis 27(3):422–431. 10.1038/s41391-024-00829-9Epub 2024 Apr 8. PMID: 38589645; PMCID: PMC11319196 38589645 10.1038/s41391-024-00829-9 PMC11319196

[16] Barros AC, Sampaio Mde C (2012) Gynecomastia: physiopathology, evaluation and treatment. Sao Paulo Med J 130(3):187–197. 10.1590/s1516-31802012000300009PMID: 22790552; PMCID: PMC10876201 22790552 10.1590/S1516-31802012000300009 PMC10876201

[17] Wilson JD, Aiman J, MacDonald PC (1980) The pathogenesis of gynecomastia. Adv Intern Med 25:1–32 PMID: 6987837 6987837

[18] Mathur R, Braunstein GD (1997) Gynecomastia: pathomechanisms and treatment strategies. Horm Res.;48(3):95–102. 10.1159/000185497. PMID: 11546925 10.1159/000185497 11546925

[19] Fagerlund A, Cormio L, Palangi L, Lewin R, Santanelli di Pompeo F, Elander A, Selvaggi G (2015) Gynecomastia in patients with prostate cancer: A systematic review. PLoS ONE 10(8):e0136094. 10.1371/journal.pone.0136094PMID: 26308532; PMCID: PMC4550398 26308532 10.1371/journal.pone.0136094 PMC4550398

[20] Cuhaci N, Polat SB, Evranos B, Ersoy R, Cakir B, Gynecomastia (2014) Clinical evaluation and management. Indian J Endocrinol Metab 18(2):150–158. 10.4103/2230-8210.129104PMID: 24741509; PMCID: PMC3987263 24741509 10.4103/2230-8210.129104 PMC3987263

[21] Sieber PR (2007) Treatment of bicalutamide-induced breast events. Expert Rev Anticancer Ther.;7(12):1773-9. 10.1586/14737140.7.12.1773. PMID: 18062751 10.1586/14737140.7.12.1773 18062751

[22] Swerdloff RS, Ng JCM, Gynecomastia: Etiology, Diagnosis (2023) and Treatment. Jan 6. In: Feingold KR, Anawalt B, Blackman MR, Boyce A, Chrousos G, Corpas E, de Herder WW, Dhatariya K, Dungan K, Hofland J, Kalra S, Kaltsas G, Kapoor N, Koch C, Kopp P, Korbonits M, Kovacs CS, Kuohung W, Laferrère B, Levy M, McGee EA, McLachlan R, New M, Purnell J, Sahay R, Shah AS, Singer F, Sperling MA, Stratakis CA, Trence DL, Wilson DP, editors. Endotext [Internet]. South Dartmouth (MA): MDText.com, Inc.; 2000–. PMID: 25905330

[23] Tyrrell CJ (1999) Gynaecomastia: aetiology and treatment options. Prostate Cancer Prostatic Dis.;2(4):167–171. 10.1038/sj.pcan.4500314. PMID: 12496773 10.1038/sj.pcan.4500314 12496773

[24] Di Lorenzo G, Perdonà S, De Placido S, D’Armiento M, Gallo A, Damiano R, Pingitore D, Gallo L, De Sio M, Autorino R (2005) Gynecomastia and breast pain induced by adjuvant therapy with bicalutamide after radical prostatectomy in patients with prostate cancer: the role of tamoxifen and radiotherapy. J Urol.;174(6):2197– 203. 10.1097/01.ju.0000181824.28382.5c. PMID: 16280763 10.1097/01.ju.0000181824.28382.5c 16280763

[25] Fradet Y, Egerdie B, Andersen M, Tammela TL, Nachabe M, Armstrong J, Morris T, Navani S (2007) Tamoxifen as prophylaxis for prevention of gynaecomastia and breast pain associated with bicalutamide 150 mg monotherapy in patients with prostate cancer: a randomised, placebo-controlled, dose-response study. Eur Urol.;52(1):106– 14. doi: 10.1016/j.eururo.2007.01.031. Epub 2007 Jan 16. PMID: 17270340 10.1016/j.eururo.2007.01.031 17270340

[26] Saltzstein D, Sieber P, Morris T, Gallo J (2005) Prevention and management of bicalutamide-induced gynecomastia and breast pain: randomized endocrinologic and clinical studies with tamoxifen and anastrozole. Prostate Cancer Prostatic Dis.;8(1):75–83. 10.1038/sj.pcan.4500782. PMID: 15685254 10.1038/sj.pcan.4500782 15685254

[27] Serretta V, Altieri V, Morgia G, Nicolosi F, De Grande G, Mazza R, Melloni D, Allegro R, Ferraù F, Gebbia V (2012) A randomized trial comparing tamoxifen therapy vs. tamoxifen prophylaxis in bicalutamide-induced gynecomastia. Clin Genitourin Cancer.;10(3):174-9. doi: 10.1016/j.clgc.2012.03.002. Epub 2012 Apr 12. PMID: 22502790 10.1016/j.clgc.2012.03.002 22502790

[28] Ozen H, Akyol F, Toktas G, Eskicorapci S, Unluer E, Kuyumcuoglu U, Abay E, Cureklibatur I, Sengoz M, Yalcin V, Akpinar H, Zorlu F, Sengor F, Karaman I (2010) Is prophylactic breast radiotherapy necessary in all patients with prostate cancer and gynecomastia and/or breast pain? J Urol 184(2):519–524 Epub 2010 Jun 17. PMID: 20620411 20620411 10.1016/j.juro.2010.03.137

[29] Tyrrell CJ, Payne H, Tammela TL, Bakke A, Lodding P, Goedhals L, Van Erps P, Boon T, Van De Beek C, Andersson SO, Morris T, Carroll K (2004) Prophylactic breast irradiation with a single dose of electron beam radiotherapy (10 Gy) significantly reduces the incidence of bicalutamide-induced gynecomastia. Int J Radiat Oncol Biol Phys.;60(2):476– 83. 10.1016/j.ijrobp.2004.03.022. PMID: 15380582 10.1016/j.ijrobp.2004.03.022 15380582

[30] Widmark A, Fosså SD, Lundmo P, Damber JE, Vaage S, Damber L, Wiklund F, Klepp O (2003) Does prophylactic breast irradiation prevent antiandrogen-induced gynecomastia? Evaluation of 253 patients in the randomized Scandinavian trial SPCG-7/SFUO-3. Urology.;61(1):145– 51. 10.1016/s0090-4295(02)02107-6. PMID: 12559286 10.1016/s0090-4295(02)02107-6 12559286

[31] Ghadjar P, Aebersold DM, Albrecht C, Böhmer D, Flentje M, Ganswindt U, Höcht S, Hölscher T, Müller AC, Niehoff P, Pinkawa M, Sedlmayer F, Zips D, Wiegel T, Prostate Cancer Expert Panel of the German Society of Radiation Oncology (DEGRO), The Working Party Radiation Oncology of the German Cancer Society (DKG-ARO) (2020) Treatment strategies to prevent and reduce gynecomastia and/or breast pain caused by antiandrogen therapy for prostate cancer: statement from the DEGRO working group prostate cancer. Strahlenther Onkol 196(7):589–597. 10.1007/s00066-020-01598-9Epub 2020 Mar 12. PMID: 32166452; PMCID: PMC7305090 32166452 10.1007/s00066-020-01598-9 PMC7305090

[32] Tunio MA, Al-Asiri M, Al-Amro A, Bayoumi Y, Fareed M (2012) Optimal prophylactic and definitive therapy for bicalutamide-induced gynecomastia: results of a meta-analysis. Curr Oncol 19(4):e280–e288. 10.3747/co.19.993PMID: 22876157; PMCID: PMC3410840 22876157 10.3747/co.19.993 PMC3410840

[33] Moher D, Shamseer L, Clarke M, Ghersi D, Liberati A, Petticrew M, Shekelle P, Stewart LA, PRISMA-P Group (2015) Preferred reporting items for systematic review and meta-analysis protocols (PRISMA-P) 2015 statement. Syst Rev 4(1):1. 10.1186/2046-4053-4-1 25554246 10.1186/2046-4053-4-1 PMC4320440

[34] Jadad AR, Moore RA, Carroll D, Jenkinson C, Reynolds DJ, Gavaghan DJ, McQuay HJ (1996) Assessing the quality of reports of randomized clinical trials: is blinding necessary? Control Clin Trials 17(1):1–12 8721797 10.1016/0197-2456(95)00134-4

[35] Higgins JP, Thompson SG (2002) Quantifying heterogeneity in a meta-analysis. StatMed 21:1539–1558 10.1002/sim.1186 12111919

[36] Sterne JA, Egger M, Smith GD (2001) Systematic reviews in health care: investigating and dealing with publication and other biases in meta-analysis. BMJ 323(7304):101–105. 10.1136/bmj.323.7304.101PMID: 11451790; PMCID: PMC1120714 11451790 10.1136/bmj.323.7304.101 PMC1120714

[37] Duval SJ, Tweedie RL (2000) A non-parametric trim and fill method of assessing publication bias in meta-analysis. J Am Stat Assoc 95:89–98

[38] Parker C, Castro E, Fizazi K, Heidenreich A, Ost P, Procopio G, Tombal B, Gillessen S, ESMO Guidelines Committee (2020) Prostate cancer: ESMO clinical practice guidelines for diagnosis, treatment and follow-up. Ann Oncol 31(9):1119–1134. 10.1016/j.annonc.2020.06.011 32593798 10.1016/j.annonc.2020.06.011

[39] Cornford P, Tilki D, van den Bergh RCN et al (2024) EAU-EANM-ESTRO-ESUR-ISUP-SIOG Guidelines on Prostate Cancer– Limited Update April 2024. *Eur Urol*. Available at: https://uroweb.org/guideline/prostate-cancer. Accessed March 12, 2025

[40] Tsuboi I, Schulz RJ, Laukhtina E, Wada K, Karakiewicz PI, Araki M, Shariat SF (2025) Incidence, management, and prevention of gynecomastia and breast pain in patients with prostate cancer undergoing antiandrogen therapy: A systematic review and Meta-analysis of randomized controlled trials. Eur Urol Open Sci 73:31–42 PMID: 39935942; PMCID: PMC11810703 39935942 10.1016/j.euros.2025.01.001 PMC11810703

[41] Huggins C, Hodges CV (1941) Studies on prostatic cancer. II. The effects of castration on advanced carcinoma of the prostate gland. Arch Surg 43(5):709–723

[42] Han BH, Lim JS, Kim SW (2013) Low-dose radiotherapy in the treatment of gynecomastia induced by antiandrogen therapy. Int J Radiat Oncol Biol Phys 87(5):950–956

[43] Santen RJ, Mansel R, Goldstein L (2010) The role of aromatase inhibitors in gynecomastia prevention. Lancet Oncol 11(10):855–864

[44] Bedognetti D, Rubagotti A, Conti G, Francesca F, De Cobelli O, Canclini L, Gallucci M, Aragona F, Di Tonno P, Cortellini P, Martorana G, Lapini A, Boccardo F (2010) An open, randomised, multicentre, phase 3 trial comparing the efficacy of two Tamoxifen schedules in preventing gynaecomastia induced by bicalutamide monotherapy in prostate cancer patients. Eur Urol 57(2):238–245. 10.1016/j.eururo.2009.05.019Epub 2009 May 19. PMID: 19481335 19481335 10.1016/j.eururo.2009.05.019

